# ASSESSING THE POTENTIAL OF DIGITAL HOME ASSISTANTS TO SUPPORT OLDER ADULTS WITH MOBILITY DISABILITY

**DOI:** 10.1093/geroni/igac059.1215

**Published:** 2022-12-20

**Authors:** Kenneth Blocker, Ki Lim, Husna Hussaini, Kyreon Williams, Ramavarapu S Sreenivas, Wendy Rogers

**Affiliations:** University of Illinois Urbana-Champaign, Champaign, Illinois, United States; University of Illinois Urbana-Champaign, Champaign, Illinois, United States; University of Illinois Urbana-Champaign, Champaign, Illinois, United States; university of Illinois Urbana-Champaign, Champaign, Illinois, United States; University of Illinois Urbana-Champaign, Champaign, Illinois, United States; University of Illinois Urbana Champaign, Champaign, Illinois, United States

## Abstract

Digital home assistants (e.g., Amazon Echo devices) hold great potential for supporting older adults in completing a wide range of daily living activities and improving their overall quality of life. As these devices can wirelessly connect with other smart technologies to increase their capabilities (e.g., environmental control), digital assistance technologies may benefit older adults who need support within their home such as those living with mobility disabilities. However, research is needed to better understand the facilitators and barriers of using these technologies by these individuals, as well as the resources that best support their onboarding and continued use. To assess the potential efficacy of integrating smart and connected technologies into the homes of older adults with mobility disability, we are conducting a field trial to understand how best to introduce these devices and support their use. Design considerations and initial findings are discussed.

